# Systematic Analysis of Galactinol Synthase and Raffinose Synthase Gene Families in Potato and Their Expression Patterns in Development and Abiotic Stress Responses

**DOI:** 10.3390/genes14071344

**Published:** 2023-06-26

**Authors:** Quankai Jing, Airu Chen, Zhaoyan Lv, Zhihao Dong, Lixia Wang, Xiaoke Meng, Yue Feng, Yu Wan, Chengyun Su, Yanjie Cui, Wenjuan Xu, Hualan Hou, Xiaobiao Zhu

**Affiliations:** School of Horticulture, Anhui Agricultural University, Hefei 230000, China

**Keywords:** potato, abiotic stress, galactinol synthase, raffinose synthase, gene expression pattern

## Abstract

Raffinose family oligosaccharides (RFOs) are very important for plant growth, development, and abiotic stress tolerance. Galactinol synthase (GolS) and raffinose synthase (RFS) are critical enzymes involved in RFO biosynthesis. However, the whole-genome identification and stress responses of their coding genes in potato remain unexplored. In this study, four *StGolS* and nine *StRFS* genes were identified and classified into three and five subgroups, respectively. Remarkably, a total of two *StGolS* and four *StRFS* genes in potato were identified to form collinear pairs with those in both Arabidopsis and tomato, respectively. Subsequent analysis revealed that *StGolS4* exhibited significantly high expression levels in transport-related tissues, PEG-6000, and ABA treatments, with remarkable upregulation under salt stress. Additionally, *StRFS5* showed similar responses to *StGolS4*, but *StRFS4* and *StRFS8* gene expression increased significantly under salt treatment and decreased in PEG-6000 and ABA treatments. Overall, these results lay a foundation for further research on the functional characteristics and molecular mechanisms of these two gene families in response to ABA, salt, and drought stresses, and provide a theoretical foundation and new gene resources for the abiotic-stress-tolerant breeding of potato.

## 1. Introduction

Potato (*Solanum tuberosum* L.) is globally recognized as the foremost non-cereal food crop, and it belongs to the genus *Solanum* of family *Solanaceae* [1,2]. It is widely cultivated and essential for food security around the world [3]. Regrettably, potato production is frequently plagued by an array of abiotic stresses, with salt and drought stresses representing the primary factors that restrict yields. Salt stress inhibits tuber growth and causes yield losses of up to 60% in potato [4]. In a recent study, 103 commercial potato cultivars were found to have a decreased fresh tuber weight by 54% under drought stress, with the cultivar Connantre showing the greatest reduction [5,6]. Therefore, improving the salt and drought tolerance of potato is a viable pathway to ensure agricultural sustainability.

Some osmolytes, including proline, mannitol, trehalose, and raffinose, are commonly accumulated in various plant species in response to different abiotic stresses [7,8]. Raffinose family oligosaccharides (RFOs) are small and soluble molecules that serve various functions in plant biology. They act as primary transport carbohydrates in some plant species, serve as signaling molecules after pathogen attack and physical injury, and accumulate in vegetative tissues as a response to diverse abiotic stresses [9]. The accumulation of raffinose is considered as an important indicator of the plant stress response, particularly under drought and salt stresses. Several studies have shown that drought and salt stresses significantly increase raffinose levels [10,11]. Some researchers propose that raffinose’s natural hygroscopic properties might protect plants from drought stress [12]. Additionally, the accumulation of raffinose in plants was closely linked to enhanced antioxidant capacity [13]. This relationship has significant implications as it can effectively mitigate the oxidative damage induced by drought and salt stresses. The identification of the crucial genes involved in raffinose biosynthesis in potato under drought and salt stresses is of paramount importance, given the substantial role of raffinose in plant responses to abiotic stress.

The galactinol pathway assumes a fundamental role in the biosynthesis of RFOs. Extra galactose molecules contribute to sucrose through the addition of galactinol, which results in the synthesis of more complex RFOs, including raffinose [14]. Galactinol synthase (GolS, EC: 2.4.1.123) plays a vital and indispensable role in the initial and critical steps in the formation of the raffinose biosynthetic pathway [15]. Multiple *GolS* genes have been documented to be induced by stress stimuli and displayed a significant positive correlation with the plant’s resistance to various abiotic stresses. *Arabidopsis*, for instance, possesses a total of seven *GolS* genes, with *AtGolS1* and *AtGolS2* responding to drought and salt stresses, while *AtGolS3* specifically responds to cold stress [11]. Remarkably, overexpression of the *AtGolS2* gene in rice has been demonstrated to significantly enhance drought tolerance in transgenic plants [16]. Arabidopsis transgenic lines with overexpressed chickpea *GolS* genes (*CaGolS1* and *CaGolS2*) demonstrated that *CaGolS* enhanced dehydration stress tolerance by modulating ROS scavenging [15]. Similarly, overexpression of the wheat *GolS3* gene (*TaGolS3*) in transgenic Arabidopsis and rice has been found to regulate ROS production, thereby enhancing the tolerance of these plants to zinc stress [17]. In maize, *ZmGolS2* was highly expressed in germinating seeds under dehydration stress, while *ZmGolS3* mainly accumulated in seeds before maturation drying [18].

RFOs are synthesized through the transfer of galactose from galactinol to sucrose under the catalysis of raffinose synthase (RFS, EC 2.4.1.82), finally producing trisaccharide raffinose [19]. Studies on *RFS* genes are relatively less common than those on *GolS* genes in plants, but recent studies have unveiled the pivotal roles of *RFS* genes in enhancing plant tolerance to abiotic stresses. Several abiotic-stress-induced RFSs (RS-1 to -6) exist in *Arabidopsis thaliana* (*A. thaliana*), and one abiotic-stress-induced RFS isoform (RS5, At5g40390) was found to be responsible for raffinose biosynthesis in leaves [20]. The maize *RFS* gene (*ZmRAFS*) exhibited heightened expression under salt, drought, heat, and cold stresses, which enhanced plant drought tolerance through the synthesis of raffinose or the hydrolysis of galactinol [21]. The raffinose content increased significantly in maize leaves when the *ZmRAFS* gene was overexpressed, and the drought tolerance was enhanced in plants [12].

Abscisic acid (ABA), a plant hormone, exerts regulatory control over various aspects of plant physiology and metabolism, particularly in abiotic stress conditions [22,23]. The accumulation of raffinose oligosaccharides, particularly stachyose, was induced by ABA, while the activity and transcript levels of galactinol synthase, raffinose synthase, and stachyose synthase were increased in somatic embryos of alfalfa [24]. The ABA content and the transcripts of genes involved in the biosynthesis of stress-protective metabolites, such as raffinose, trehalose, and proline, were found to increase under combined stress in maize [25]. This suggests that an important role is played by ABA-dependent transcriptional control in the plant’s acclimation to adverse conditions. During cold acclimation in grapevine woody tissues, ABA accumulation led to the upregulation of *VivRafS5*, resulting in raffinose synthesis [26]. Therefore, unraveling the effect of ABA on the expression of key genes *StGolSs* and *StRFSs*, pivotal in potato raffinose biosynthesis, holds critical significance in elucidating the potato’s response to abiotic stress.

The advent of new sequencing technologies has greatly facilitated the whole-genome research of numerous organisms, enabling the exploration of gene families. Although the *GolS* and *RFS* gene families have been revealed in many plant species, a comprehensive analysis of these genes in potato, especially in response to ABA, salt, and drought stresses, has not been reported. In this study, we conducted a comprehensive investigation aiming to identify the *StGolS* and *StRFS* genes in the potato genome, and their phylogenetic relationships, gene structures, conserved motifs, chromosomal locations, and expression patterns under ABA, salt, and drought stresses were systematically analyzed. The results obtained from this study lay a foundation for further research on the functional characteristics and molecular mechanisms of the *StGolS* and *StRFS* genes in response to ABA, salt, and drought stresses in potato, and they provide a theoretical basis and new gene resources for the development of potato varieties tolerant to abiotic stress.

## 2. Materials and Methods

### 2.1. Plant Material and Abiotic Stress Treatments

The doubled monoploid potato clone DM1-3 516 R44 (DM, 2*n* = 2*x* = 24) was used in this study and its tissue-cultured plants were grown in test tubes with 10 mL Murashige and Skoog (MS) solid medium containing 3% (*w/v*) sucrose (pH 5.6~5.8). The photoperiod in the light incubator for DM tissue-cultured plants was 16 h daylight at 22 °C and 8 h darkness at 18 °C [27,28].

The concentrations of NaCl, PEG-6000, and abscisic acid (ABA) in the treatments were the same as those in previous studies [29,30,31]. Here, 45-day-old test tube plantlets were used for ion balance with 10 mL MS liquid medium with or without 400 mM NaCl (the final concentration of NaCl was 200 mM) for 24 h, and subsequently all of the liquid media were removed immediately from the test tubes. The aboveground parts of the test tube plantlets with 200 mM NaCl treatment were sampled at 3 h (S1), 6 h (S2), 12 h (S3), and 24 h (S4) after the 24 h ion balance, and the 24 h samples without 200 mM NaCl treatment (after the 24 h ion balance with 10 mL MS liquid medium) were considered as the controls (CK). All of the collected samples were placed into liquid nitrogen immediately and stored at −80 °C.

Similar to the previous methods, 30% PEG-6000 + MS liquid medium (simulating drought stress) and 0.2 mM abscisic acid + MS liquid medium (ABA) were added to the test tube plantlets, respectively. After balancing for 24 h, the liquid medium was removed. At 3 h, 6 h, 12 h, and 24 h (P1, P2, P3, and P4; A1, A2, A3, and A4), the aboveground parts of the test tube plantlets were collected and stored at −80 °C. The 24 h samples without any stress (after the 24 h ion balance with 10 mL MS liquid medium) were the controls (CK). Each experiment was repeated three times with three biological replicates and four test tube plantlets for each replicate.

### 2.2. Identification of the StGolS and StRFS Genes

The whole-genome data used in this study were downloaded from the Spud DB (http://spuddb.uga.edu, accessed on 11 January 2022), which contains the newest potato genome DM v6.1 [32]. Amino acid sequences of the previously identified genes from *Arabidopsis* were used to perform a BLASTp search to obtain homologs with an E-value cutoff of 10^−5^ [33]. The profile hidden Markov model of the HMMER3.0 program was applied to verify the identification results [34]. The amino acid sequence information of each gene was further verified by the Pfam [35] and SMART [36] online tools. The physicochemical properties of each protein, including the molecular weight (MW), theoretical isoelectric point (pI), and hydrophilicity mean (GRAVY), were calculated by the online tool ProtParam [37].

### 2.3. Chromosome Localization of the StGolS and StRFS Genes

The position information of potato genes on chromosomes can be obtained from the Spud DB. Based on the relative position of each gene on each chromosome, the chromosome localization map of the genes was drawn using the Mapinspect software (http://www.softsea.com/download/MapInspect.html, accessed on 11 January 2022).

### 2.4. Phylogenetic Tree Construction of the StGolS and StRFS Genes

Using the MEGA7.0 software, amino acid sequences from potato, Arabidopsis, and tomato were aligned by Clustal W, and a phylogenetic tree was generated based on the neighbor-joining method (NJ) [38]. The phylogenetic tree was drawn and beautified using the iTOL software (https://itol.embl.de/, accessed on 3 April 2023).

### 2.5. Structure Drawing and Motif Analysis of the StGolS and StRFS Genes

Gene structure diagrams were drawn using the software TBtools [39]. The conserved functional motifs of the genes were predicted by the MEME software [40].

### 2.6. Cis-Element Analysis in Promoters of the StGolS and StRFS Genes

In order to identify the *cis*-elements in the promoters of the *StGolS* and *StRFS* genes, 1500-bp sequences before the initiation codons (ATG) were extracted from the potato genome database. We submitted the promoter sequences to the website of the PlantCARE database and analyzed the types, positions, and numbers of *cis*-elements [41,42]. The results were visualized using the software TBtools [39].

### 2.7. Syntenic Analysis of the GolS and RFS Genes

For the gene synteny among different genomes, we selected potato, tomato, and *A. thaliana* genomes for syntenic analysis. The assemblies of tomato and *A. thaliana* were obtained from TAIR (http://www.arabidopsis.org, accessed on 3 April 2023) and Phytozome (http://www.phytozome.net, accessed on 3 April 2023), respectively. The determination of syntenic blocks among potato, tomato, and *A. thaliana* was performed using the TBtools software [39].

### 2.8. RNA-seq Analysis

The potato RNA-seq data from the Spud DB (http://spuddb.uga.edu/data/DM_1-3_516_R44_potato.v6.1.TPM_gene_expression_matrix.xlsx, accessed on 3 April 2023) were used to investigate the expression characteristics of the two family genes in different tissues, including leaf, tuber, stamen, flower, shoot tip, stem, stolon, petiole, and root. The gene expression values (TPM) were presented in a heatmap, which was generated using the software TBtools [39].

### 2.9. qRT-PCR Analysis

Gene expression analysis was conducted using the quantitative real-time PCR (qRT-PCR) technique, as described previously [43,44]. Total RNA was extracted using the RNAprep Pure Plant Plus Kit (DP441, TianGen, Beijing, China), following the manufacturer’s instructions, and was reverse-transcribed into cDNA using the PrimeScript™ RT reagent Kit with gDNA Eraser (RR047A, Takara, Dalian, China). Subsequently, qRT-PCR was performed in triplicate for each sample on the CFX96 Touch^TM^ Real-Time PCR Detection System (Bio-Rad, Washington, DC, USA) using the TB Green^®^ Premix Ex Taq™ I (RR820A, Takara, Dalian, China). The reference gene *StActin97* was used to normalize the gene expression levels. The relative expression levels of the genes were calculated using the Gene Expression Macro software, version 1.1 (Bio-Rad Laboratories, Washington, DC, USA). The sequences of the specific primers used for qRT-PCR are listed in Supplementary Table S1.

## 3. Results

### 3.1. Identification of the GolS and RFS Gene Families in Potato

Based on the amino acid sequences of the *AtGolS* and *AtRS* genes, a comprehensive analysis was conducted to identify the *GolS* and *RFS* genes in potato. The identification process involved the utilization of SMART and Pfam, which confirmed the presence of conserved domains. Four *GolS* genes were identified and designated as *StGolS1* to *StGolS4*, while nine *RFS* genes were identified and labeled as *StRFS1* to *StRFS9* (Table 1).

Subsequently, an in-depth investigation was carried out to assess the physicochemical properties of the *StGolS* and *StRFS* genes. The analysis revealed that the StGolS proteins exhibited variations in protein length, molecular weight, and theoretical isoelectric point (pI), spanning a range of 322 to 348 aa, 36.74 to 39.68 kDa, and 5.44 to 6.67, respectively (Table 1). Conversely, the StRFS proteins demonstrated a wide range of amino acid lengths, ranging from 110 to 874 aa, with molecular weights varying between 12.51 and 97.37 kDa. Notably, all StGolS and StRFS proteins exhibited acidic characteristics, as indicated by pI values below 7.00.

### 3.2. Chromosomal Distribution of the StGolS and StRFS Genes

**Figure 1:**
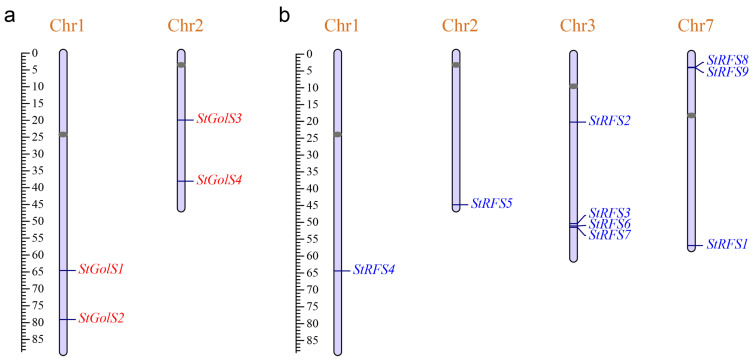
Distribution of the (**a**) *StGolS* and (**b**) *StRFS* genes on potato chromosomes. The scale bar on the left indicates the length (Mb) of the potato chromosome. The gray oval on the chromosome bar represents the centromere location for each chromosome.

The genomic distribution of the identified *StGolS* genes in potato was examined, revealing their presence on two distinct chromosomes (Figure 1a and Table 1). Specifically, genes *StGolS1* and *StGolS2* were found on chromosome 1, while genes *StGolS3* and *StGolS4* were located on chromosome 2. These results provide valuable insights into the spatial arrangement of the *StGolS* genes within the potato genome. In the case of the *StRFS* genes, their distribution across the 12 chromosomes of the potato genome was found to be non-uniform (Figure 1b and Table 1). Four *StRFS* genes were identified on chromosome 3, indicating a relatively higher density in this region. In contrast, the number of *StRFS* genes observed on chromosomes 1 and 2 was minimal, with only one gene present on each of these chromosomes. Chromosome 7 exhibited an intermediate pattern, housing three *StRFS* genes. These findings shed light on the heterogeneous distribution of the *StRFS* genes throughout the potato genome.

### 3.3. Phylogenetic Analysis of the GolS and RFS Genes from Several Differnet Species

**Figure 2:**
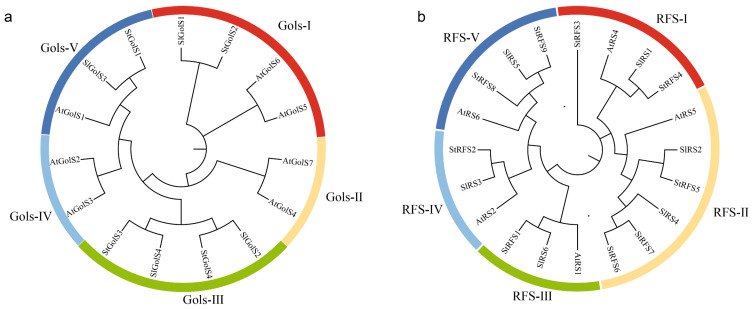
Phylogenetic tree of (**a**) GolS and (**b**) RFS proteins identified in potato, tomato, and Arabidopsis. Different subfamilies of GolS and RFS proteins were highlighted by arcs with distinct colors.

A phylogenetic analysis was conducted to investigate the relationships of *GolS* and *RFS* family genes among three different plant species, namely potato, tomato [45,46], and Arabidopsis [47]. The protein sequences of four *StGolSs*, seven *AtGolSs*, and four *SlGolSs* were aligned, and a phylogenetic tree was constructed (Figure 2a and Supplementary Materials, Table S2). Based on their homology relationships, the four StGolS proteins could be classified into three subgroups. *StGolS2* and *SlGolS1* belonged to subgroup I and showed homology with *AtGolS5* and *AtGolS6*, respectively. The branches of the phylogenetic tree indicated a close genetic relationship between the potato and tomato *GolS* genes in subgroup III, which contained *StGolS3* and *StGolS4*. Subgroup V comprised *StGolS1* and *SlGolS3*, which displayed homology with *AtGolS1*. Subgroups II and IV consisted exclusively of AtGolS proteins, with no corresponding proteins found in potato and tomato.

For the *RFS* genes, multiple sequence alignment was performed on the protein sequences from potato, tomato, and Arabidopsis, followed by the construction of a neighbor-joining tree using MEGA7.0 (Figure 2b and Supplementary Materials Table S2). The *RFS* genes were categorized into five distinct groups based on their homology relationships. Group I encompassed *StRFS3*, *StRFS4*, and *SlRS1*, which exhibited homology with *AtRS4*. Group II included *StRFS5*, *StRFS6*, *StRFS7*, *SlRS2*, and *SlRS4*, showing homology with *AtRS5*. *StRFS1* and *SlRS6* comprised group III, displaying homology with *AtRS1*. Group IV consisted of *StRFS2* and *SlRS3*, which exhibited homology with *AtRS2*. Finally, group V was composed of *StRFS8*, *StRFS9*, and *SlRS5*, demonstrating homology with *AtRS6*.

### 3.4. Gene Structure and Motif Composition of the StGolS and StRFS Genes

**Figure 3:**
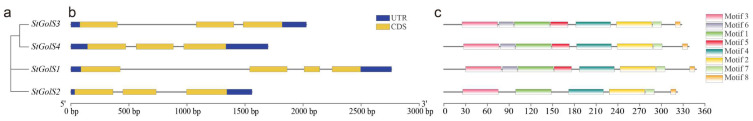
Gene structure of *StGolS* genes and conserved motif analysis of StGolS proteins. (**a**) Gene phylogenetic tree; (**b**) gene structure; and (**c**) protein conserved motifs of StGolS proteins.

**Figure 4:**
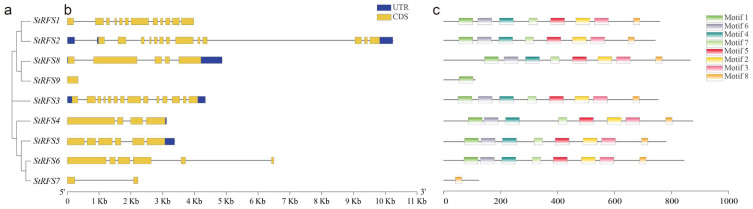
Gene structure of *StRFS* genes and conserved motif analysis of StRFS proteins. (**a**) Gene phylogenetic tree; (**b**) gene structure; and (**c**) protein conserved motifs of StRFS proteins.

The arrangement of exons and introns plays a crucial role in the evolution of gene families [48]. A phylogenetic tree was constructed using full-length protein sequences (Figure 3 and Figure 4) to establish relationships between gene sequences and their corresponding exon/intron distributions and motif compositions. Gene structure and motif analyses of the *StGolS* and *StRFS* genes were performed to gain insights into their evolutionary characteristics.

Regarding the *StGolS* genes, there was observed variation in the exon/intron organization across different members of the family (Figure 3a,b). Specifically, *StGolS1* consisted of four exons, whereas the remaining three *StGolS* genes contained three exons within their coding regions. In order to obtain a deeper understanding of the diversity of StGolS proteins, conserved motifs were identified using the MEME program. A total of eight conserved motifs were discovered, with motif 8 being the shortest and consisting of eight aa (Supplementary Materials, Table S3). The distribution of these motifs varied among different subgroups (Figure 3c). Motifs 1, 2, 3, 4, 7, and 8 were found to be shared among all *StGolS* members, suggesting their conserved nature. On the other hand, motifs 5 and 6 were deficient in *StGolS2*.

The exon–intron architecture of the *StRFS* genes was thoroughly investigated by aligning their genomic and coding sequences, as depicted in Figure 4a,b. The comprehensive analysis unveiled a pattern wherein the majority of *StRFS* genes exhibited multiple introns, ranging from 3 to 13, with the sole exception of *StRFS7*, which possessed a single intron. A distinct characteristic was observed in *StRFS9*, which lacked introns entirely and solely comprised exonic regions within their coding sequences. This analysis provides valuable insights into the structural organization of the *StRFS* genes. A total of eight conserved motifs were detected (Figure 4c and Supplementary Materials, Table S4). Notably, except for StRFS7 and StRFS9, all other StRFS proteins encompassed the aforementioned eight identified motifs. StGolS9 exclusively contained motif 1, while StGolS7 only possessed motif 8. This meticulous motif analysis contributes valuable information concerning the conservation and potential functional elements within the StRFS proteins.

### 3.5. Cis-Element Analysis in Promoters of the StGolS and StRFS Genes

**Figure 5:**
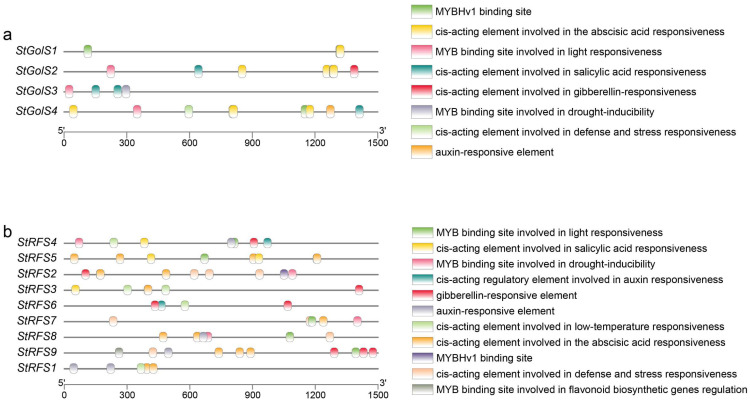
Distribution of *cis*-acting elements in (**a**) *StGolS* and (**b**) *StRFS* gene promoters.

To gain further insights into the potential functions of the *StGolS* and *StRFS* genes in potato, an analysis of the *cis*-acting elements within their promoter regions was conducted. For the *StGolS* genes, *cis*-acting elements were predicted in the 1500-bp sequences upstream of the transcription start site (TSS). Some significant *cis*-acting elements were identified in the promoters of the *StGolS* genes, including four elements associated with plant hormone responses, three MYB binding sites, and one *cis*-acting element involved in defense and stress responses (Figure 5a). It should be noted that, except for the *StGolS3* gene, multiple *cis*-acting elements involved in abscisic acid responsiveness were found in the promoter regions of all *StGolS* genes, suggesting their potential involvement in ABA-mediated processes. The promoter of the *StGolS4* gene contained the highest abundance of *cis*-acting elements, with a maximum of nine, encompassing six different types. Within the *cis*-acting elements of the *StGolS4* gene, there were four *cis*-acting elements involved in abscisic acid responsiveness and one auxin-responsive element.

A total of 61 *cis*-elements were detected in the promoter regions of the *StRFS* genes, belonging to five hormone-related elements, four MYB-related sites, and two stress-related elements (Figure 5b). The hormone-related elements encompassed an ABA response element, salicylic acid (SA) response element, gibberellin (GA) response element, and two auxin response elements. Among the hormone-related elements, the *cis*-acting element involved in abscisic acid responsiveness was the most abundant, with a total of 18, distributed in the promoter regions of seven *StRFS* genes, excluding *StRFS4* and *StRFS6*. Furthermore, *cis*-acting elements involved in defense and stress responsiveness were found in the promoter regions of the *StRFS2*, *StRFS7*, *StRFS8*, and *StRFS9* genes. The *cis*-element analysis provides valuable information regarding the potential regulatory mechanisms and functional implications of the *StGolS* and *StRFS* genes in potato, particularly their involvement in hormone responses and stress-related processes.

### 3.6. Interspecific Synteny of the GolS and RFS Genes from Several Different Species

**Figure 6:**
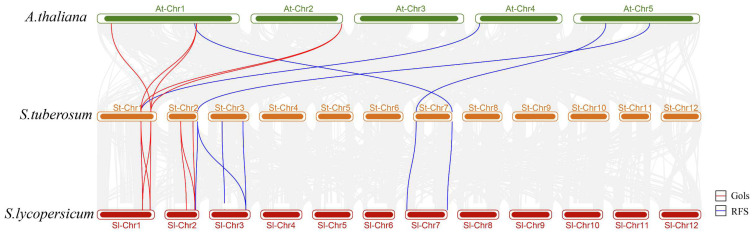
Syntenic relationships of the *GolS* and *RFS* genes among Arabidopsis, potato, and tomato. Redlines indicate the homology and evolutionary links of *GolS* genes, and blue lines indicate the homology and evolutionary links of *RFS* genes. Gray lines indicate all the syntenic blocks present in their respective genomes. The Arabidopsis chromosome was represented by the green bars, while the potato chromosome was represented by the orange bars, and the tomato chromosome was represented by the red bars.

To further explore the evolutionary relationship between the *GolS* and *RFS* genes in potato and the genes in Arabidopsis and tomato, synteny analysis was performed. The results demonstrated a syntenic relationship between four of the *StGolS* genes and the *GolS* genes from tomato, followed by two *StGolS* genes with Arabidopsis (Figure 6 and Supplementary Materials, Tables S5 and S6). Additionally, a syntenic relationship was observed between six *StRFS* genes and the *RFS* genes from tomato, along with four *StRFS* genes and Arabidopsis (Figure 6 and Tables S5 and S6). Remarkably, a total of two *StGolSs* (*StGolS1* and *StGolS2*) and four *StRFSs* (*StRFS1*, *StRFS4*, *StRFS5*, and *StRFS8*) in potato were identified to form collinear pairs with those in both Arabidopsis and tomato, respectively. The number of collinear pairs of *StGolS* and *StRFS* genes localized at highly conserved syntenic regions from tomato, belonging to the *Solanum* family, was greater than that from Arabidopsis, belonging to the dicot species. These results underscore the homology among the *GolS* and *RFS* genes of Arabidopsis, potato, and tomato, thus implying potential functional similarities in their respective gene functions.

### 3.7. Expression Characteristics of the StGolS and StRFS Genes in Different Tissues of Potato

**Figure 7:**
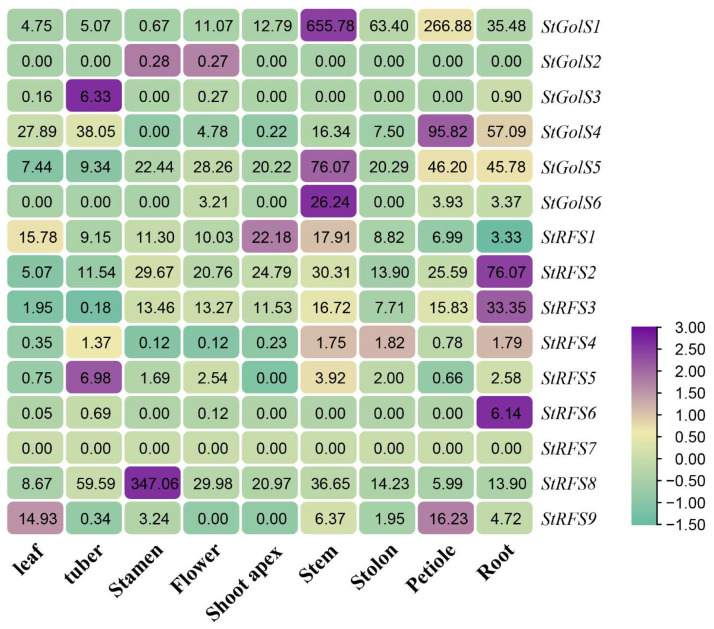
Expression profiles of the *StGolS* and *StRFS* genes in different potato organs and tissues.

To gain insights into the expression characteristics of the *StGolS* and *StRFS* family genes across different tissues (leaf, tuber, stamen, flower, shoot tip, stem, stolon, petiole, and root), we analyzed their gene expression patterns using potato RNA-seq data available in the Spud database (Figure 7). Among the *StGolS* genes, *StGolS2* showed minimal expression throughout development, while *StGolS3* displayed only slight expression in tubers. Regarding transport-related tissues (stem, petiole, and root), *StGolS1* and *StGolS4* were prominently expressed, whereas the expression of other genes in this family was comparatively weak.

Among the nine *StRFS* genes, *StRFS4* and *StRFS7* displayed relatively low expression levels across all tissues. *StRFS5* and *StRFS6* were solely expressed in tubers and roots, while *StRFS9* showed expression in leaves and petioles. *StRFS1*, *StRFS2*, and *StRFS8* exhibited high expression levels in all tissues (Figure 7). By elucidating the expression profiles of the *StGolS* and *StRFS* genes in diverse tissues, our analysis provides valuable insights into their tissue-specific functions in potato development.

### 3.8. Expression Patterns of the StGolS and StRFS Genes in Potato under Abiotic Stress

**Figure 8:**
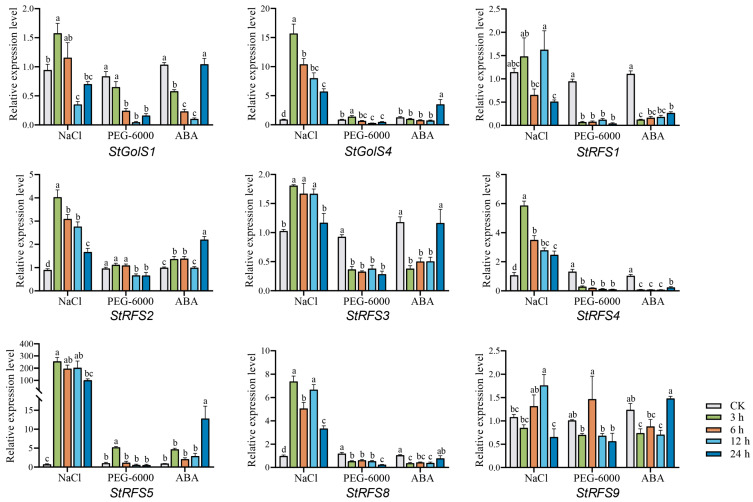
Relative expression levels of the *StGolS* and *StRFS* genes in DM test tube plantlets under salinity (NaCl), drought (PEG-6000), and ABA treatment. Gene expression is shown relative to the expression of the reference gene *StActin97*. Bars represent mean ± standard error (SE) of three biological replicates. Statistical significance was analyzed by the ANOVA at *p* < 0.05, lowercase letters indicate significant differences among different samples from each treatment and genes.

To investigate the involvement of the *StGolS* and *StRFS* genes in the response to abiotic stress, we collected qRT-PCR data from various treatments, including salinity, drought, and abscisic acid (ABA), at different time points (3 h, 6 h, 12 h, and 24 h). Importantly, despite similar sequences within subfamilies, not all genes exhibited identical expression levels (Figure 8). Under salt stress conditions, *StGolS1*, *StGolS4*, *StRFS2*, *StRFS3*, *StRFS4*, *StRFS5*, *StRFS8*, and *StRFS9* showed significant upregulation. In the presence of PEG-6000, *StGolS4* and *StRFS5* displayed notable increases in their expression levels. Likewise, the expression of *StGolS4*, *StRFS2*, and *StRFS5* was prominently upregulated in response to ABA treatment. Furthermore, we observed that the expression levels of *StGolS4* and *StRFS5* were significantly elevated after exposure to NaCl, PEG-6000, and ABA. Additionally, salt treatment led to a significant increase in the expression of *StRFS4* and *StRFS8*, while PEG-6000 and ABA treatments resulted in decreased expression. Genes with low expression levels (Ct > 35) across all treatments were excluded from the analysis. The expression patterns of the *StGolS* and *StRFS* genes in response to salinity, drought, and ABA treatments shed light on their potential roles in mediating plant adaptation to abiotic stresses.

## 4. Discussion

RFOs have various functions, such as seed maturation and transporting and storing sugars, and are considered to be related to plant drought and salt tolerance. The enzymes galactinol synthase (GolS) and raffinose synthase (RFS) are crucial for the biosynthesis of raffinose, a key component of RFOs. However, there is limited knowledge regarding the *GolS* and *RFS* gene families in potato, making it imperative to investigate these genes from a comprehensive whole-genome perspective. Our results lay a foundation for further study of the functional characteristics and molecular mechanisms of the *StGolS* and *StRFS* genes in response to ABA, salt, and drought stresses in potato.

### 4.1. Analysis of the Number, Structure, and Evolution of the GolS and RFS Genes in Potato

Variation in the number of genes may be related to the evolutionary diversity, genome duplication events, and genome sizes in plants [49]. In potato, only four *StGolS* genes located on two chromosomes were identified, and the number of these genes is comparatively lower than in both kiwifruit [50] and sesame [46], but is consistent with findings in tomato [45]. The structural disparities in exons and introns may arise from specific splicing events and serve as informative markers in investigating their evolutionary mechanisms [51]. The observed limited variation in the exon–intron structures of the *StGolS* genes, with three to four exons across different species, aligns with previous reports in other plants [49,52,53]. Furthermore, the four *StGolS* genes can be classified into three subfamilies, which aligns with the findings in tomato [45]. In this study, nine *StRFS* genes were found to be distributed on four chromosomes in potato, consistent with the findings in the tea genome [54]. Phylogenetic analysis revealed that all *StRAFS* genes could be categorized into five subgroups. Amino acid and nucleotide sequence analysis indicated that members of the *StGolS* and *StRFS* gene families within the same subgroup exhibited similar structural features and conserved motifs, suggesting a close evolutionary relationship, as reported in sesame [46].

### 4.2. StGolS and StRFS Genes Play a Key Role in Potato Growth and Development

The expression patterns of *GolS* genes in different tissues of potato indicate that different GolS proteins may fulfill distinct functions. Previous studies have linked *GolS* expression in leaves to phloem export, carbon storage, and plant defense against abiotic stress and oxidative damage [55]. Specifically, the orthologs of *Pa×gGolSI* [56] and members of the *BnGolS3* subfamily [57] were found to be highly expressed in leaves. In contrast, no significant expression of *GolS* genes was observed in tobacco leaves [57]. Additionally, the expression of *GolS* in the root tissues has been linked to the response to osmotic stress [58]. In our study, *StGolS4* displayed high expression levels in transport-related tissues, suggesting that *StGolS4* may play a crucial role in material transport in potato. The expression profiles of the *StRFS* genes in different tissues were also examined using RNA-seq data. In cotton, *GhRFS1A*, *GhRFS2D*, *GhRFS8A*, and *GhRFS8D* displayed consistently high expression levels across all tissues, indicating their potential role in promoting vegetative growth in upland cotton [51]. In the case of potato, *StRFS1*, *StRFS2*, and *StRFS8* exhibited high expression levels, suggesting their significance during potato growth. In addition, relatively low expression levels were observed for *StRFS4* across all tissues, while *StRFS5* exhibited exclusive expression in tubers.

### 4.3. Differential Expression of Potato StGolS and StRFS Genes under Salinity, Drought, and Abscisic Acid

Raffinose plays important roles in plant seed development and participates in the plant response to different external stresses [51]. The raffinose content increases significantly in plants in response to abiotic stress [59]. In this study, the expression levels of several *StGolS* and *StRFS* genes were found to be upregulated under salinity, drought, and abscisic acid (ABA) treatments. Studies on *GolS* genes in Verbascum phoeniceum [60] and cassava [49] have shown different expression characteristics under salt and drought stresses, suggesting that each member of the gene family may have distinct physiological roles. The induction of *GolS* genes by salt stress has also been observed in other plants, such as *AtGolS1* in Arabidopsis [11] and *StGolS4* in the same subgroup, which displayed significant increases in expression under salt treatment. Phylogenetic analysis revealed that *StRFS2*, *StRFS5*, and *StRFS8*, which exhibited high expression levels under salt stress in potato, belonged to three distinct subgroups. The results suggest that not all *StRFS* genes within the same subgroup and with similar sequences exhibit the same expression levels.

*StGolS4* and *StRFS5* were found to be activated under salinity, drought, and ABA treatments. The upregulation of these genes in response to NaCl treatment may be induced by osmotic stress caused by salt. Furthermore, due to its high expression in transport-related tissues, it is highly likely that *StGolS4* plays a key role in potato material transport and osmotic regulation. Its upregulated expression under salt stress might be attributed to the influence of osmotic stress induced by salt. Notably, an analysis of the promoter region of *StGolS4* revealed the existence of ABA-responsive motifs, which are regulated by ABA. While the expression levels of *StRFS4* and *StRFS8* significantly increased under salt stress, they decreased under PEG-6000 and ABA treatment, suggesting that their responses to salt stress may involve other mechanisms, such as ion toxicity.

## 5. Conclusions

In this study, a total of four *StGolS* and nine *StRFS* genes were identified in the whole genome of DM potato and were systematically analyzed by bioinformatics, and their expression patterns in different tissues, under ABA and abiotic stresses, were investigated. The two gene families were classified into three and five subgroups, respectively. Remarkably, a total of two *StGolS* and four *StRFS* genes in potato were identified to form collinear pairs with those in both Arabidopsis and tomato, respectively. *StGolS4* exhibited significantly high expression levels in transport-related tissues and in PEG-6000 and ABA treatments, with remarkable upregulation under salt stress. Similar to *StGolS4*, *StRFS5* exhibited comparable responses to the treatments. Intriguingly, *StRFS4* and *StRFS8* demonstrated increased expression under salt treatment but decreased expression in PEG-6000 and ABA treatments. Taken together, the results lay a foundation for further study of the functional characteristics and molecular mechanisms of the *StGolS* and *StRFS* genes in response to ABA, salt, and drought stresses in potato, and they provide a theoretical basis and new gene resources for the development of potato varieties with tolerance to abiotic stresses.

## Figures and Tables

**Table 1:** *StGolS* and *StRFS* genes identified in potato and their sequence characteristics.

Gene Name	Gene ID	Chromosome	Start (bp)	End (bp)	Exon Number	Protein Length (aa)	MolWt (kDa)	pI
*StGolS1*	Soltu.DM.01G025230	chr01	64,586,706	64,589,468	4	348	39.68	6.67
*StGolS* *2*	Soltu.DM.01G040570	chr01	79,121,977	79,123,537	3	322	36.74	6.40
*StGolS3*	Soltu.DM.02G006360	chr02	19,943,641	19,945,670	3	328	37.56	5.61
*StGolS4*	Soltu.DM.02G024820	chr02	38,094,421	38,096,119	3	338	38.65	5.44
*StRFS1*	Soltu.DM.07G027830	chr07	56,852,708	56,856,677	12	757	83.82	6.19
*StRFS2*	Soltu.DM.03G008410	chr03	20,239,428	20,249,663	14	742	81.85	6.44
*StRFS3*	Soltu.DM.03G025220	chr03	50,422,790	50,427,128	14	752	83.26	5.59
*StRFS4*	Soltu.DM.01G025080	chr01	64,419,085	64,422,201	4	874	97.37	5.67
*StRFS5*	Soltu.DM.02G033230	chr02	44,798,199	44,801,571	6	780	86.87	5.97
*StRFS6*	Soltu.DM.03G026160	chr03	51,135,322	51,141,812	6	843	94.30	5.97
*StRFS7*	Soltu.DM.03G026620	chr03	51,449,668	51,451,882	2	123	13.90	4.12
*StRFS8*	Soltu.DM.07G003310	chr07	3,899,124	3,903,987	5	865	94.91	5.24
*StRFS9*	Soltu.DM.07G003360	chr07	3,954,988	3,955,317	1	110	12.51	6.52

## Data Availability

The data supporting the findings of this study are available within the article and its Supplementary Materials.

## Supplementary Materials

The following supporting information can be downloaded at: https://www.mdpi.com/article/10.3390/genes14071344/s1. Table S1. Primers for qRT-PCR; Table S2. Most conserved eight motifs of StGolS proteins; Table S3. Most conserved eight motifs of StRFS proteins; Table S4. *GolS* and *RFS* genes in representative plant species; Table S5. Syntenic gene pairs between *S. tuberosum* and *A. thaliana*; Table S6. Syntenic gene pairs between *S. tuberosum* and *S. lycopersicum*.
