# Supplementary material for: Systematic Analysis of Galactinol Synthase and Raffinose Synthase Gene Families in Potato and Their Expression Patterns in Development and Abiotic Stress Responses
Source: Genes (Basel). 2023 Jun 26;14(7):1344. doi: 10.3390/genes14071344 (PMC10379439; doi:10.3390/genes14071344)
Supplement: Supplementary file 1 [file genes-14-01344-s001.zip › genes-2446789-supplementary.pdf]

Table S1. Primers for qRT-PCR.

Gene Name	Forward Primer (5′-3′)	Reverse Primer (5′-3′)	Product Length
StActin97	AGTATGACGAATCTGGTCCTTCTATTG	ACCCAACAATCAACTCTGCCCTCTC	203 bp
StGolS1	GGGTCCTGCTACATTGGACC	AATCTCCCTCACGACACAGC	196 bp
StGolS2	TTGCTGAGCAGGACTTTCTGA	CCCAGTGTATTTCCACGGCT	173 bp
StGolS3	TTAGTTTTGGCTATGTTGTGGC	CCGTCCAATTTTTCACTAGCAT	160 bp
StGolS4	AGGCGAAATCCGCTTATCCA	TCCACAAACTCCCAAATACGGA	193 bp
StRFS1	TCCTGCAATGGGACTTCGAC	GTCCCGGGAGATGAAACTGG	171 bp
StRFS2	GGATCAGTACTTCGCGCTCA	GTTGTAAGTGTGCCTGGGGA	209 bp
StRFS3	CAAAAAGGTGCCAGGACATTTA	AGACCTTCTTTAATCCCCTGTG	93 bp
StRFS4	TGGCACCACCAAATGATCCA	ATCCGCCTTTGGAAGACGTT	228 bp
StRFS5	TAACTTCCACTCCGGGTTGC	GGCCGGCCTAATGAATCAGA	214 bp
StRFS6	TTCCTGCCAACATCATTGCC	GCCTACCCAATGAGTGGTCC	176 bp
StRFS7	ATGTATAGCCAGAAGAAACTAAAGCTCT	TCCAATGCTTACTGAACTTTCTTCC	234 bp
StRFS8	AGGCCAATCCAAAACCCCAT	CCGGAGTAATCGTCATGGCA	226 bp
StRFS9	GCGATCATCACTCCAACGA	TGTCTTGATCGAATTCTGCACC	157 bp

Table S2. *GolS* and *RFS* genes in representative plant species.

Species	Name	Locus	Data Source
A. thaliana	*AtGolS1*	At2g47180	TAIR
	AtGolS2	At1g56600	
	AtGolS3	At1g09350	
	AtGolS4	At1g60470	
	AtGolS5	At5g23790	
	AtGolS6	At4g26250	
	AtGolS7	At1g60450	
Solanum lycopersicum	*SlGolS1*	Solyc01g100830	Phytozome
	SlGolS2	Solyc02g084980	
	SlGolS3	Solyc01g079170	
	SlGolS4	Solyc02g062590	
A. thaliana	*AtRS1*	AT1G55740	TAIR
	AtRS2	AT3G57520	
	AtRS4	AT4G01970	
	AtRS5	AT5G40390	
	AtRS6	AT5G20250	
Solanum lycopersicum	*SlRS1*	Solyc01g079300	Phytozome
	SlRS2	Solyc02g086530	
	SlRS3	Solyc03g058970	
	SlRS4	Solyc03g112500	
	SlRS5	Solyc07g007930	
	SlRS6	Solyc07g065980	

Data source: TAIR, the Arabidopsis Information Resource, http://www.arabidopsis.org, accessed on 3 April 2023; Phytozome, http://www.phytozome.net, accessed on 3 April 2023.

Table S3. Eight most conserved motifs of StGolS proteins.

Motif	Width (aa)	Sequences
1	50	VINYSKLRIWEFVEYSKMIYLDGDIQVFDNIDHLFDLPDGYFYAVMDCFC
2	50	AMLWRHPENVEJDKVKVVHYCAAGSKPWRYTGKEENMDREDIKMLVKKWW
3	50	TFLAGNGDYVKGVVGLAKGLRKVKSAYPLVVAVLPDVPEEHRRILINQGC
4	49	YFNAGMFVFZPNLSTYDDLLKTLKVTPPTPFAEQDFLNMYFKDIYKPIP
5	25	EKTWSHTPQYKIGYCQQCPDKVQWP
6	21	VREIEPVYPPENQTQFAMAYY
7	13	DIYNDESLDYKNS
8	8	YITAPSAA

Table S4. Eight most conserved motifs of StRFS proteins.

Motif	Width (aa)	Sequences
1	50	SHHVVPJGKLQDIRFMSJFRFKLWWMTQRMGTSGSDIPMETQFLJLEVKD
2	50	TIHIAHVAYNSLWMGEFIQPDWDMFQSTHPCAEFHAAARAISGGPVYVSD
3	50	PDGSILRAQLPGRPTRDCLFEDPLRDGKSLLKIWNLNKYSGVIGAFNCQG
4	50	PGIVDWFGWCTWDAFYLTVNPZGVKEGLESLVEGGCPPRFVJIDDGWQSI
5	50	LHSYLASAGIDGVKVDVQHJLETLGEGYGGRVELAKKYYQALEASIAKNF
6	50	FLPJJEGSFRAVLQGGKBDELEICVESGDSKVKTSSFSEIVYMHAGDDPF
7	30	KEFHGLKYVYVWHALCGYWGGVRPGVPGME
8	23	QFAPIGLVBMLNSGGAIEGLEYE

Table S5. Syntenic gene pairs between *S. tuberosum* and *A. thaliana.*

Gene Name	Gene ID of *S. tuberosum*	Gene ID of *A. thaliana*
StGolS1	Soltu.DM.01G025230	AT1G56600
StGolS1	Soltu.DM.01G025230	AT2G47180
StGolS2	Soltu.DM.01G040570	AT1G56600
StGolS2	Soltu.DM.01G040570	AT1G09350
StGolS2	Soltu.DM.01G040570	AT2G47180
StRFS1	Soltu.DM.07G027830	AT1G55740
StRFS4	Soltu.DM.01G025080	AT4G01970
StRFS5	Soltu.DM.02G033230	AT5G40390
StRFS8	Soltu.DM.07G003310	AT5G20250

Table S6. Syntenic gene pairs between *S. tuberosum* and *S. lycopersicum.*

Gene Name	Gene ID of *S. tuberosum*	Gene ID of *S. lycopersicum*
StGolS1	Soltu.DM.01G025230	Solyc01T003408
StGolS1	Soltu.DM.01G025230	Solyc01T002086
StGolS2	Soltu.DM.01G040570	Solyc01T003408
StGolS2	Soltu.DM.01G040570	Solyc01T002086
StGolS3	Soltu.DM.02G006360	Solyc02T000743
StGolS3	Soltu.DM.02G006360	Solyc02T002113
StGolS4	Soltu.DM.02G024820	Solyc02T002113
StGolS4	Soltu.DM.02G024820	Solyc02T000743
StRFS1	Soltu.DM.07G027830	Solyc07T002763
StRFS2	Soltu.DM.03G008410	Solyc03T001161
StRFS4	Soltu.DM.01G025080	Solyc01T002098
StRFS5	Soltu.DM.02G033230	Solyc02T002236
StRFS5	Soltu.DM.02G033230	Solyc03T002506
StRFS6	Soltu.DM.03G026160	Solyc03T002506
StRFS8	Soltu.DM.07G003310	Solyc07T000298
